# 41 Cases of Treatment of Cranial Cruciate Ligament Rupture with Porous TTA: Three Years of Follow Up

**DOI:** 10.3390/vetsci6010018

**Published:** 2019-02-20

**Authors:** Rodrigo Trisciuzzi, Laura Fracassi, Hernando Afonso Martin, Donato Monopoli Forleo, Daniel Amat, Leonor Santos-Ruiz, Elena De Palma, Alberto Maria Crovace

**Affiliations:** 1 Department of Emergencies and Organ Transplantation (DETO), University of Bari, Aldo Moro, 70010 Bari, Italy; rodrigo.trisciuzzi@uniba.it (R.T.); laura.fracassi@uniba.it (L.F.); depalma.elena@gmail.com (E.D.P.); 2 PHD Course of Transplantation of Tissue and Organs and Cell Therapy (DETO) University of Bari, Aldo Moro, 70126 Bari, Italy; 3 Istituto Tecnologico de Canaria (ITC), Canary Islands, 35003 Las Palmas, Spain; hafonso@itccanarias.org (H.A.M.); dmonopoli@itccanarias.org (D.M.F.); 4 Center for Biomedical Research in Network, Bioengineering, Biomaterials and Nanomedicine (CIBER-BBN), Carlos III Health Institute, 28029 Madrid, Spain; mr_amat@hotmail.com (D.A.); lsantos@uma.es (L.S.-R.); 5 Departamento de Biología Celular, Genética y Fisiología, IBIMA-Universidad de Málaga, Facultad de Ciencias, Campus de Teatinos, 29071 Málaga, Spain; 6 PHD Course of Health and Veterinary Experimental Sciences, University of Perugia, 06123 Perugia, Italy

**Keywords:** tibial tuberosity advancement, titanium porous cage, dogs

## Abstract

Tibial Tuberosity Advancement (TTA) is a surgical technique based on a linear osteotomy that determines a cranial advancement of the tibial tuberosity in patients suffering from cranial cruciate ligament rupture (CCL). The aim is to neutralize the cranial tibial thrust (CTT) and to reach a 90° angle between the patellar tendon and the tibial plateau with a physiological knee extension of 135°. In our study, a Ti6AI4V ELI (Titanium Aluminium Vanadium) titanium scaffold for the Porous TTA, with excellent properties of osteointegration and osteoconduction when subjected to cyclic loading has been adopted. Based on the previous scientific work on an ovine model, the use of this type of porous scaffolds has subverted the previous models. Scaffold production technology is based on direct mechanical manufacturing called Electron Beam Melting (EBM). For this study, 41 dogs, different breeds, medium-large size, weighing between 10 and 80 kg, aged between 1 and 13 years, were enrolled. The inclusion criteria were based on clinical evaluations (different gaits), drawer test and tibial compression, LOAD score (Liverpool Osteoarthritis in Dogs questionnaire), radiographic diagnosis in sedation with a 135° positioning of the joint and baropodometric investigations (*Stance Analyzer*). The results show that Porous TTA is an excellent method for functional recovery of the knee joint following the partial and total rupture of the CCL.

## 1. Introduction

Tibial Tuberosity Advancement (TTA) is one of the most frequently used techniques in veterinary orthopaedics to resolve cranial cruciate rupture in dogs. This technique was introduced in 2002 in veterinary medicine by Montavon, Damur and Tepic [1]. From 2002, many authors described different techniques variations using different kinds of implants to obtain the cranial advancement of the tibial tuberosity [2,3,4,5].

Some variations include the possibility to replace the original cage with other implants of different materials to improve biocompatibility and osteointegration [6].

Some authors describe the use of 3D printed customized bioceramics cages (brushite, monetite and tricalcium phosphate, and a highly permeable porous morphology) or even the use of a designed cage bio manufactured by a 3D powder printing of tricalcium phosphate cement that tailored permeability and mechanical properties.

Currently, one of the most used biomaterials for implants is titanium due to his biocompatibility and resistance to the corrosive activities of the body and its specific force related to its traction force and rigidity [7]. In the presence of oxygen, titanium gets coated by an oxide layer that increases its biocompatibility [8,9]. This characteristic makes the titanium alloy ideal for bone substitution.

Among the different titanium formulations, extra low interstitial Ti6AI4V is the most common titanium alloy used for implant manufacturing. The 3D computer designed scaffolds can be faithfully reproduced by several manufacturing techniques based on powder-bed methods using laser beam (Selective Laser Melting, SLM) or electron beam (Electron Beam Melting, EBM) [10].

Scaffold properties, such as porosity, porous size, permeability, stiffness and geometry can influence the success of implants. These parameters can be controlled to confer the mechanical device with properties more similar to the bone.

Permeability allows for the transport of nutrients and growth factor through the porous implant [11].

Porosity allows for the colonization of the scaffold by blood vessels, encouraging the growth of new bone tissue.

A porous implant has a lower stiffness compared to the solid one. This feature reduces the stress shielding effect that can occur in the site of the scaffold insertion [12,13].

Regarding geometry, different types of porous titanium structures were investigated for bone tissue engineering; between these, a particular structure, called triply periodic minimal surfaces (TPMS) has proven to be suitable for scaffolds design [14,15]. The gyroid structure is one of the most important TPMS, and due to its strength, stiffness and permeability, represents a good solution to its use for scaffolds in the reconstruction of bone defect [16].

The aim of the study was to describe the surgical technique and retrospectively evaluate feasibility and the 3-year clinical outcome, of the use of a Highly porous gyroid Ti6AI4V scaffold, sintered at Istituto Tecnologico de Canaria (ITC, Canary Islands, Las Palmas, Spain), for the stabilization of the tibial tuberosity advancement in 41 dogs naturally affected by cranial cruciate rupture. Moreover, the osteoconductivity of Ti6Al4V scaffold was evaluated in vitro. Our hypothesis was that the scaffold would guarantee a long-term clinical efficacy also due to an effective osteoconductivity.

## 2. Materials and Methods

### 2.1. Ethical Statement

The study doesn’t have an experimental nature since it is not randomized, and the technique and equipment used are available in practice since several years. Our study had a retrospective nature evaluating the 3 years clinical outcome of 41 cases performed at our institution and therefore based the legislation of the country where the study has been performed an approval form the ethical committee is not required.

### 2.2. Case Selection

All 41 patients included in this study were referred for assessment of rear lameness. All dogs belonged to canine species of different breeds of medium-large size, with a weight between 10 and 80 kg, aged between 1 and 13 years. The inclusion criteria in the study and subsequent follow-up investigations were based on a complete clinical evaluation to identify the origin of the lameness.

### 2.3. Clinical Procedures

**Figure 1:**
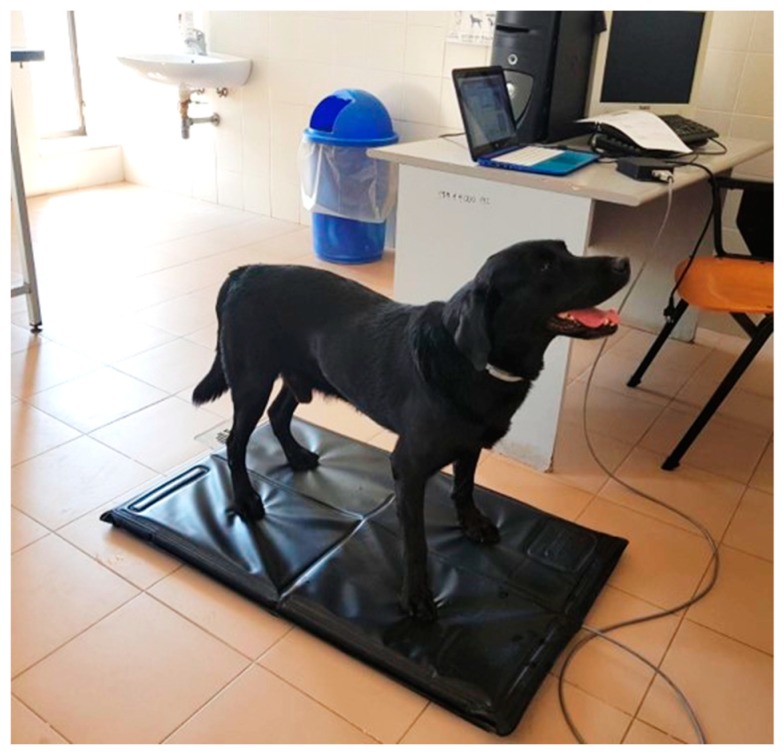
The Pet Safe Stance Analyzer. A Labrador Retriever affected by cranial cruciate ligament undergoing baropodometric evaluation standing on a padded platform. Data are elaborated by dedicated software on the main server.

We performed an orthopaedic evaluation of patients (different gaits) and functional tests of the knee joint (sit test, drawer test and tibial compression test). The score obtained by a LOAD questionnaire (Liverpool Osteoarthritis in Dogs) [17] had a score between 0–52, where 0 to 10 indicated mild pain; 11 to 20, an intermediate pain; 21 to 30, a severe pain; and 31 to 52, the presence of extreme pain. Using the analysis of load distribution onto the Stance Analyzer (Figure 1), we studied the body weight distribution on each limb, which leads to identifying a loss of load bearing [18].

### 2.4. Preoperative Planning

**Figure 2:**
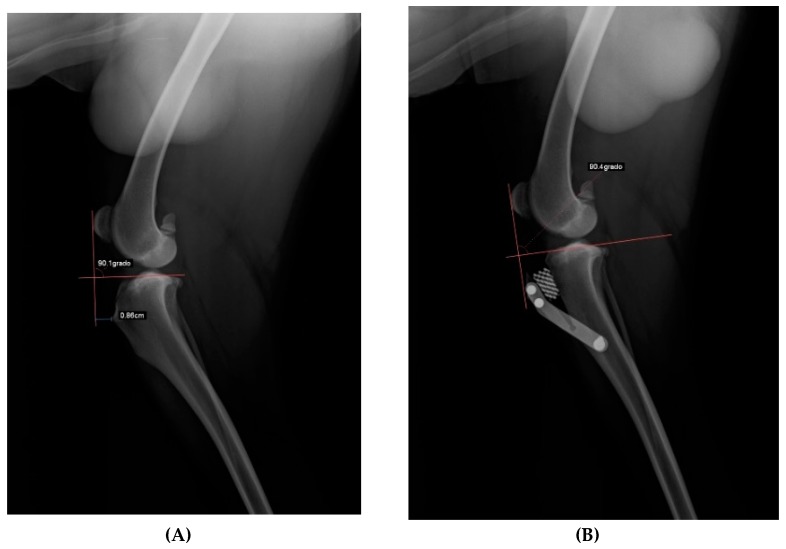
The radiographic planning. (**A**) Pre-operative measurement of the tibial advancement. (**B**) Radiographic aspect after surgery and post-operative measurement.

After acquiring clinical diagnosis, dogs were submitted to x-ray evaluation with the injured stifle with a flexion of 135° during total anaesthesia to evaluate the advancement of the tibial tuberosity with an x-ray machine (Philosophy HF 400-IPS Medical, Bussolengo, Italy), adopting the Common Tangent Method and Tibial plateau angle inclination method with the dedicated DICOM software (Horos LGPL license at Horos project.org and sponsored by Nimble Co LLC d/b/a Purview in Annapolis, MD, USA) (Figure 2).

### 2.5. Implants

The cage used in this study is a highly porous wedge (REFER Ø1) sintered by Electron Beam Melting (EBM) on Ti6Al4V (Titanium-Aluminum-Vanadium alloy) powder with a particles size of 45–70 μm in Istituto Tecnologico de Canaria (ITC, Canary Islands, Las Palmas, Spain).

The fusion process which was carried out slice by slice (70 μm thick), was with a high-power electron beam source that generated the energy needed for melting a spot on the titanium powder. These are referring to values specified on the user’s guide by the ARCAM EBM manufacturer, for the machine model S12 (production year 1997) [19].

The cleaning process used was a sandblast with the particles of the same Ti6Al4V—ELI powder but with smaller dimensions (50–60 μm). All the pieces were in an ultrasound bath for 20 min at 80 °C [20].

The engineering parameters of this scaffold (Porosity 87.09%, Yang Module 278.86 MPa, Ultimate Stress 48.88 KPa/mm^2^) were chosen by deductions after reading a few existing articles available at that time on the mechanobiology of bone regeneration [21] and after preliminary animal trials on the ovine proximal stifle made by our research group [22].

These scaffolds allowed for the ingrowth of the woven bone into its pores, resulting in better osteointegration. The titanium used in the study was configured with a deformation <2% and with a pores size of about 1 mm, and the elasticity was set between the cortical and spongious bone [23,24,25,26,27].

**Figure 3:**
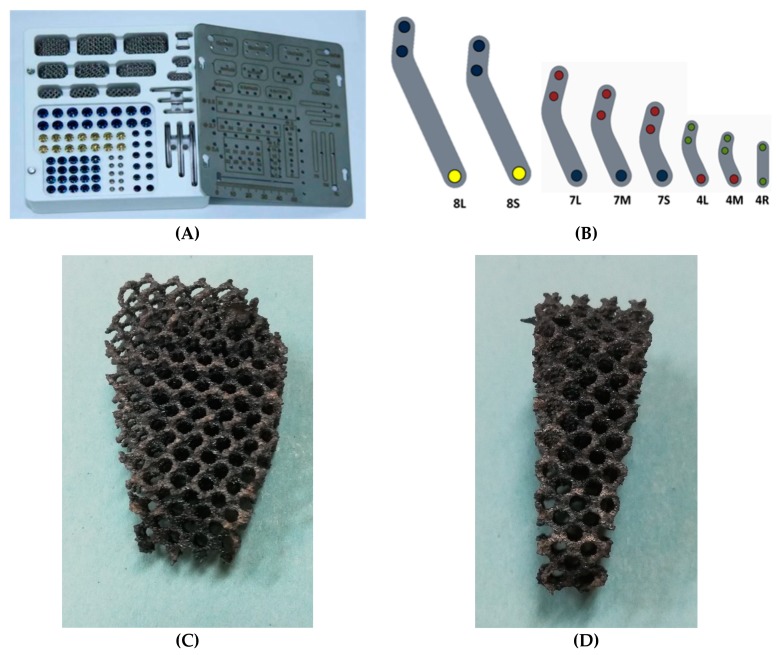
(**A**) The commercial kit with various sizes of titanium porous scaffolds, plates and screws; (**B**) Plates available within the kit; scaffolds of different size available within the kit. (**C**) Frontal view; (**D**) Lateral view. The cage and the implants were produced and developed from “Biosurgex European Union Trademark; category of Medical Instrument Products, Pharmaceutical Products” OHIM Application number 017891671.

We used commercial kits produced in ITC with titanium plates, screws and Ti6Al4V alloy cages for our patients. All those tools are present in various sizes existing for the possibility of adapting the total implant into every dog. Many sizes are available and are distinguished from height, thickness and width: sizes from 3 mm to 15 mm in terms of thickness, from 5 to 24 mm in terms of width and from 8 to 33 mm in terms of height. Sizes of the cages used in our experience were 4.5 mm (*n* = 5), 6 mm (*n* = 4), 7.5 mm (*n* = 7), 9 mm (*n* = 7), 10.5 (*n* = 13), 12 mm (*n* = 3), 15 mm (*n* = 2). Many sizes of plates are available depending on the size of the patient: 8 mm thickness plates in two different sizes (8L and 8S), 7 mm thickness plates in three different sizes (7S, 7M and 7L), and three 4 mm thickness plates in three different sizes (4S, 4M and 4L) (Figure 3).

### 2.6. Evaluation

The MC3T3-E1 pre-osteoblastic cell line, a well-established model of *in vitro* osteogenesis, was used to evaluate de osteoconductivity of Ti5Al4V scaffolds in vitro. The cells were cultured in alpha-MEM medium, supplemented with 10% Fetal Bovine Serum (FBS), 2 mM Glutamine, 100 U/mL penicillin and 100 µg/mL streptomycin [28].

Ti6Al4V porous discs, sintered by EBM, were used. Each disc had a diameter of 13 mm, a 1 mm height, and was regularly perforated by 1 mm-diameter pores. Discs were designed ad hoc to fit the wells of standard 24-well multi-well culture plates. Discs were seeded with the MC3T3-E1 cell line at a ratio of 100.000 cells/disc and allowed to grow for up to 7 days. Cell growth onto the discs was assessed by MTT assay on days 1, 3, and 7 after seeding. At the same time-points, cell viability and distribution over the disc surface was assessed, in another set of seeded discs, by Hoechst 33342 vital nuclear staining.

For MTT assays, the discs were transferred to clean wells, to avoid the interference of cells that could have been transferred from the scaffold to the bottom of the well, and covered with 1 mL culture medium containing 5 mg/mL MTT (3-(4,5-Dimethylthiazol-2-yl)-2,5-Diphenyltetrazolium Bromide). Cultures were incubated in MTT for 24 h and then the reaction was stopped by adding 1 mL of solubilizing solution (10% SDS in 0.01 M HCl), before taking three samples of the medium and measuring absorbance at 570 nm. Four cell-seeded discs were measured at each time point. Un-seeded discs were used as negative controls.

Hoechst 33342 nuclear staining was performed by incubating the cultures, for 15 min, in the culture medium 33342. Afterwards, the Hoechst medium was replaced by normal growth medium and the cultures were observed, and photographed, under epifluorescent light, with a Nikon AZ-100dissecting microscope (Nikon Instruments Europe BV, Amsterdam, Netherlands) equipped with a UV-2A filter, and a Nikon Digital Sight DS-5Mc digital camera (Nikon Instruments Europe BV, Amsterdam, Netherlands), operated by Nis-Elements software (Nikon Metrology NV—Europe, Lisses, France).

To better appreciate the shape and distribution of cells seeded on the EBM-sintered titanium discs, cells were fluorescently labelled by incubation in 1 µg/mL CMFDA-containing medium for 30 min, followed by Hoechst staining of nuclei as described. Cells were then fixed with 4% paraformaldehyde dissolved in phosphate buffered saline (PBS), for 30 min. After washing the excess paraformaldehyde with three thorough washes in PBS, actin microfilaments were fluorescently revealed with TRITC-conjugated phalloidin. For this purpose, cells were incubated for 30 min in PBS containing 50 µg/mL TRITC-phalloidin conjugate and 0.1% TRITON^®^ X-100. Afterwards, they were washed in PBS, observed and imaged with a Leica SP5 II laser confocal microscope operated by Leica LAS AF software (Leica Microsystems, Wetzlar, Germany). Three-dimensional reconstructions and animations were generated with the mentioned.

### 2.7. Surgical Procedure

Signed informed owner consent was collected for all patients to perform sedation and general anaesthesia.

**Figure 4:**
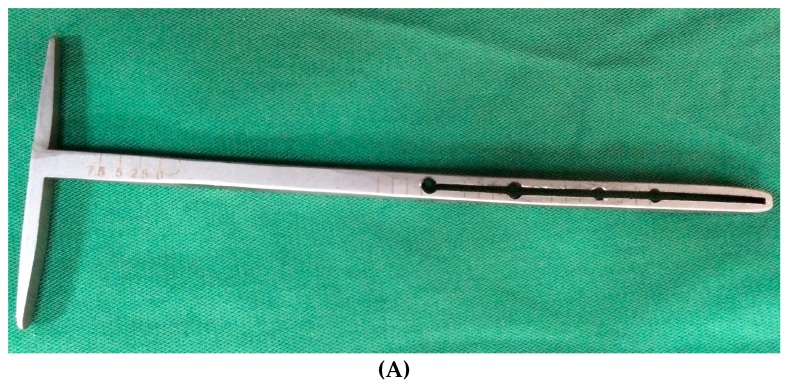

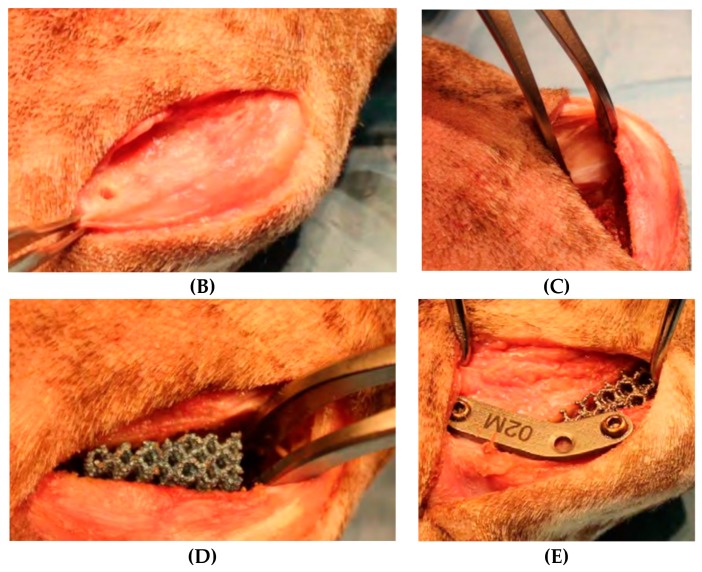
The surgical procedure (**A**) Osteotomy guide; (**B**) Maquet hole on the distal tibial tuberosity; (**C**) distraction of the tibial tuberosity; (**D**) porous titanium cage in the osteotomy; (**E**) plate and screws placed to fix the implant.

The surgery was started with dedicated instrumentation made by the ICT of Canaries, and with dogs under general anaesthesia. A Maquet hole was made on the medial aspect of the distal tibial tuberosity. With the use of a dedicated guide (Figure 4A), an osteotomy of the tibial tuberosity was made and with a distractor, this osteotomy was slowly distracted to permit the introduction of a titanium cage into the osteotomy. A proper titanium plate with three screws was applied on the cranial part of the osteotomized tibial tuberosity and on tibial diaphysis to complete the surgery.

Sutures and postoperative X-ray, to evaluate the results of the advancements, completed the procedure.

### 2.8. Radiographic and Clinical Follow-Up

Patients had the first clinical and x-ray investigation 15 days after surgery.

Mediolateral and anteroposterior radiographs of the treated limb were acquired for each patient, to evaluate the surgical procedure, implants and scaffold’s osteointegration. Then, the patients were checked with LOAD questionnaire and a Stance Analyzer monthly from the day of surgery (T0) to sixth months for the statistical analysis (T6) [29].

### 2.9. Statistical Analysis

The MedCalc^®^ software was used with the Paired sample T-Test function for the statistical evaluation of the data obtained both from the LOAD questionnaires and from the values obtained from the baropodometric analysis (MedCalc Statistical Software version 16.4.3 MedCalc Software bvba, Ostend, Belgium; https://www.medcalc.org; 2016). The P value threshold was *p* < 0.05.

## 3. Results

A total of 41 dogs, with 22 males and 19 females, with 36.6 ± 18.1 kg medium weight, 5.4 ± 3.1 years medium age, enrolled in our study. The breeds included in the study were: mixed breed (*n* = 12), Epagneul Breton (*n* = 2), Golden Retriever (*n* = 2), Turkmen Alabai (*n* = 2), Caucasian Shepherd dog (*n* = 1), German Shepherd (*n* = 1), American Staffordshire Terrier (*n* = 2), Italian Pointer (*n* = 1), Staffordshire Bull terrier (*n* = 1), Beagle (*n* = 1), Labrador Retriever (*n* = 1), American Pitt Bull Terrier (*n* = 4), Maremman Hound (*n* = 1), Cane Corso (*n* = 3), Italian Hound (*n* = 1), Shar Pei (*n* = 1), Neapolitan Mastiff (*n* = 1), Rottweiler (*n* = 1), Argentin Dogo (*n* = 1), Samoyed (*n* = 1), Boxer (*n* = 1).

### 3.1. In Vitro Results

The *in vitro* tests showed that MC3T3-E1 preosteoblastic cells adhered to the EBM-sintered titanium scaffolds and grew onto them, populating their whole surface.

**Figure 5:**
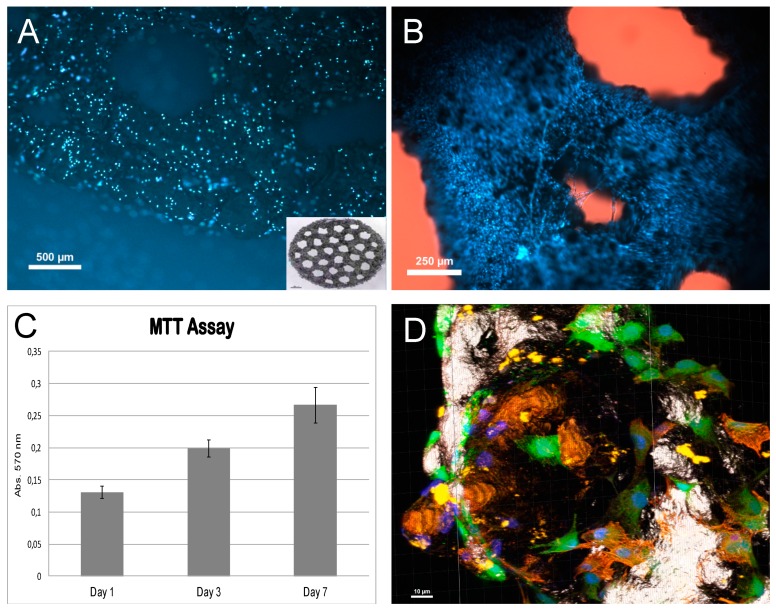
The *in vitro* evaluation. (**A**) and (**B**) Hoechst staining showing the nuclei of living cells seeded onto titanium discs at 1 day and 7 days, respectively, after seeding. Insert in (A) shows one of the discs used for the *in vitro* assays (ø ≈ 13 mm). Note the different cell density between (A) and (B). (**C**) MTT assay of the preosteoblastic cell cultures. As seen in the graph, the population size increased progressively with time. (**D**) 3D-reconstruction of confocal planes of MCT3-E1 cells stained with CMFDA (5-chloromethylfluorescein diacetate) (green), Hoechst (blue), and TRITC-phalloidin (Phalloidin–Tetramethylrhodamine B isothiocyanate) (red). The titanium surface has been given a silver-gold pseudocolour. An animation of this figure can be found as Supplementary Data, where action microfilaments and cell bodies extension can be better observed.

Hoechst staining on day 1 after seeding (Figure 5A) showed that cells had attached to the titanium surface, being evenly distributed. Staining on later days showed an increased cell density, indicating that attached cells have multiplied on the titanium. By the 7th day after seeding, the whole surface appeared to be covered up by cells (Figure 5B). These observations were confirmed by MTT assay, which showed that the cell population increase over time in the culture (Figure 5C).

To further evaluate the phenotype of osteoblastic cells on titanium, early cultures where triple-labelled with Hoechst 33352 to visualize the nuclei, vital staining (CMFDA) to observe cell cytoplasm, and actin-microfilaments staining. This allowed us to observe the cell bodies of cells after attachment. As seen in Figure 5D, cell bodies appeared extended on the titanium substrate, indicating good attachment. Many of them displayed and abundant microfilament network, and extensions of the cell body, possibly indicative of the migration over the substratum in order to colonize it.

### 3.2. Clinic Results

A percentage of 73% of patients was able to properly load the limb moments after awakening; the same percentage of dogs showed a proper recovery of the correct load 15 days after surgery and last 27% acquired a correct load bearing during further controls. A total of 100% of patients evaluated on T6 showed a total recovery and absence of lameness.

**Figure 6:**
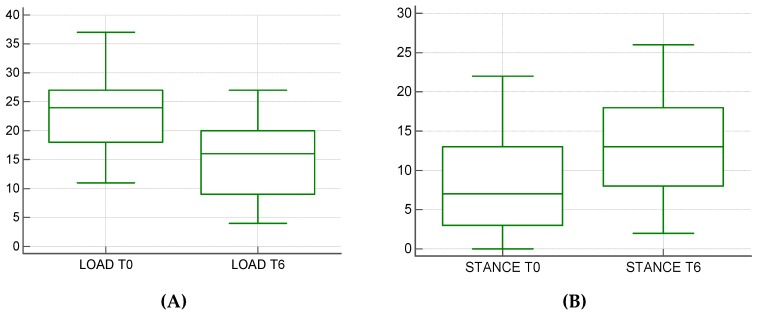
(**A**) A graphic representation of the significant decrease of the LOAD score of dogs from T0 to T6; (**B**) A graphic representation of the significant improvement of the percentage of load bearing on the injured limb from T0 to T6.

In our study, we analyzed the clinical and x-ray data, LOAD and baropodometric score, on the surgery day (T0) and six months after surgery (T6). For each patient, orthogonal radiographic projections were obtained to evaluate osteolysis signs, implant failure or scaffold-related infections. A LOAD questionnaire was administered to every owner during every control and every patient was evaluated onto the Pet Safe Stance Analyzer. The registered complications were attributed to the surgical technique adopted. In 7 of 41 dogs the fixation of the Maquet hole was recorded leading to the fracture of the tibial tuberosity. Only in one case was it necessary to apply a surgical revision to reduce the avulsion of the tibial tuberosity. In two cases, the surgical wound dehiscence was recorded. LOAD data decreased from an average of 22.88 ± 6.63 to an average of 14.5 ± 6.53, presenting a statistically significant difference (*p* < 0.0004) (Figure 6A) Of the 41 patients, 10 exams for incomplete data on baropodometric analysis were excluded. The mean load rate went from a mean average of 8.4 ± 6.38 to an average of 13.06 ± 6.85 with a statistically significant difference (*p* < 0.0034). (Figure 6B).

**Figure 7:**
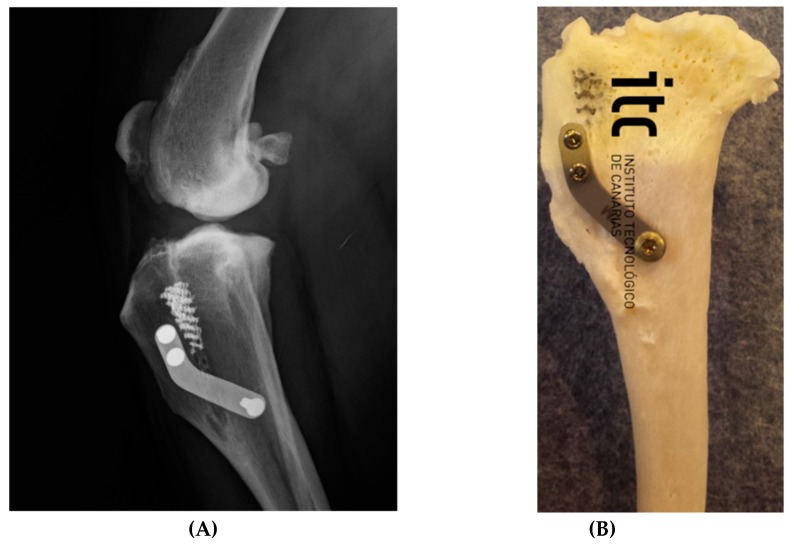
The osteointegration of porous titanium cage. (**A**) Mediolateral projection of a dog who had undergone Porous TTA. Control after three years. (**B**) Post mortem aspect of the integration of bone and scaffold (two years after surgery).

Of the 41 patients, 100% of the recruited cases showed signs of scarring of the osteotomy at T6. The first signs of new bone formation in the scaffold and in the site of osteotomy were identified after an average of 2.3 ± 1.4 months (Figure 7).

## 4. Discussion

The porous titanium scaffolds are used in many surgical fields, such as the correction of angular deviations or the regeneration of bone tissue following large losses of substance.

For the *in vitro* evaluation, porous discs and cylinders were seeded with osteoblastic cells to prove Ti-EBM alloy is osteoconductive.

In a study conducted by Yànez et Al. in 2018, the structure of Ti6Al4V alloy scaffolds was studied. Here, it is emphasized that for large bones, the defects are located mainly in the epiphyseal area, where the distribution of the load does not follow the longitudinal force lines and in the diaphysis and metaphysis areas, where there is more abundance of cortical bone and where the load is distributed in the longitudinal direction of the bone. For this reason, their applicability in orthopaedic surgery, as well as their design and characterization towards their use for the tissue reconstruction of large segmental bone defects, is claimed [16].

TTA is one of the surgical uses that has allowed us to obtain encouraging results in the follow up conducted in our experience. Over the years the TTA technique has undergone different modifications in the procedure (TTA rapid, first and second generation TTA) [30,31,32] and has been compared with common extracapsular and intracapsular surgical techniques applied to patients suffering from cranial cruciate ligament rupture, such as TPLO [33].

Here we report the experience of 41 dogs suffering from cranial cruciate ligament rupture undergoing the Porous TTA technique. The surgical procedure adopted was the modified Maquet technique [34], making a hole in the most distal point of the tibial tuberosity and progressively distracting it. Complications were not associated with the material used because there were no implant failures. In cases of fissures near the Maquet holes, no surgical revision was necessary. In fact, by positioning the distal hole of the plate distally, the hole of Maquet and on the tibial diaphysis, the implant prevents the complete avulsion of the tibial tuberosity. Clinically, more than 80% of patients presented positive clinical results after 15 days of follow-up, both in terms of LOAD score and baropodometric analysis. The complications recorded during our follow up did not require a surgical review because the stability of the implant allowed the maintenance of a positive clinical outcome in 98% of the patients. Compared with the results of previous tibial tuberosity advancement techniques, Tibial tuberosity advancement in small-breed dogs using TTA Rapid implants lead to the following conclusion: complications and outcome [35] porous TTA presented better clinical success with a percentage of complications (17%) similar to the one reported in the previous studies. In our clinical experience, the modified Maquet technique was associated with the use of high-porosity Ti6Al4V alloy scaffolds with a gyroid structure. The decision to use these implants is justified by the need to improve the healing of the osteotomy, accelerating integration times of the titanium scaffold and obtaining positive clinical results in a short time. Some authors suggest filling the osteotomy region with hydroxyapatite paste [36]. However, the use of this biomaterial proved to be unnecessary as for classical surgical TTA techniques [37] as for the Porous TTA in our experience. Moreover, the *in vitro* results confirm that the use of three-dimensional scaffolds with a porosity of 87.09%, Yang Module 278.86 MPa, Ultimate Stress 48.88 KPa/mm^2^, allows us to obtain the best colonization of the implants used and the best biomechanical adaptability. Radiographically, the first signs of osseointegration confirmed the results of the *in vitro* study, allowing the first signs of bone deposition inside the osteotomy to be appreciated as early as 60 days after surgery. This study is a practical example of how the precise design of porous material can strongly change the total performance of the system even without changing the external shape of the same. Future studies will clarify if this technique can also influence the development and progression of osteoarthritis as proven for the TPLO [38].

## 5. Conclusions

Ti6AI4V porous scaffold used for the Porous TTA can be considered a valid alternative to other devices used in TTA due to its osteoconductive and osteointegrative abilities. Moreover, all the patients reached the desired levelling of the tibial plateau thanks to the different sizes available of the scaffold and also the possibility to adapt them to the osteotomy since they are cuttable. The implant of the scaffold is simple and requires few dedicated instruments. The use of the scaffold gives good stability to the advancement since it takes advantage of the elasticity of the bone and its macroporosity allows an early formation of woven bone to obtain an armed bone at the end of the reparative process. Further investigation and aims should focus on the long-term clinical evaluation of patients undergoing surgery. A future aim should be the radiological evaluation of the bone density in the scaffold area using a dedicated CT elaboration to develop a scan levelling density scale, in order to perform a quantitative and qualitative evaluation of the woven bone into the titanium alloy scaffolds.

## Supplementary Materials

The following are available online at http://www.mdpi.com/2306-7381/6/1/18/s1, Supplementary Data: 5D-reconstruction of confocal planes of MCT3-E1 cells stained with CMFDA (5-chloromethylfluorescein diacetate) (green), Hoechst (blue), and TRITC-phalloidin (Phalloidin–Tetramethylrhodamine B isothiocyanate) (red).
